# *ty-5* Confers Broad-Spectrum Resistance to Geminiviruses

**DOI:** 10.3390/v14081804

**Published:** 2022-08-17

**Authors:** Yanxiang Ren, Xiaorong Tao, Dawei Li, Xiuling Yang, Xueping Zhou

**Affiliations:** 1 State Key Laboratory of Agro-Biotechnology, College of Biological Sciences, China Agricultural University, Beijing 100193, China; 2 State Key Laboratory for Biology of Plant Diseases and Insect Pests, Institute of Plant Protection, Chinese Academy of Agricultural Sciences, Beijing 100193, China; 3 Key Laboratory of Plant Immunity, Department of Plant Pathology, Nanjing Agricultural University, Nanjing 210095, China; 4 State Key Laboratory of Rice Biology, Institute of Biotechnology, Zhejiang University, Hangzhou 310058, China

**Keywords:** geminivirus, *ty-5*, resistance, Pelota

## Abstract

The selection of resistant crops is an effective method for controlling geminivirus diseases. *ty-5* encodes a messenger RNA surveillance factor Pelota with a single amino acid mutation (Pelota^V16G^), which confers effective resistance to tomato yellow leaf curl virus (TYLCV). No studies have investigated whether *ty-5* confers resistance to other geminiviruses. Here, we demonstrate that the tomato *ty-5* line exhibits effective resistance to various geminiviruses. It confers resistance to two representative begomoviruses, tomato yellow leaf curl China virus/tomato yellow leaf curl China betasatellite complex and tomato leaf curl Yunnan virus. The *ty-5* line also exhibits partial resistance to a curtovirus beet curly top virus. Importantly, *ty-5* confers resistance to TYLCV with a betasatellite. Southern blotting and quantitative polymerase chain reaction analyses showed that significantly less DNA of these geminiviruses accumulated in the *ty-5* line than in the susceptible line. Moreover, knockdown of *Pelota* expression converted a *Nicotiana benthamiana* plant from a geminivirus-susceptible host to a geminivirus-resistant host. Overall, our findings suggest that *ty-5* is an important resistance gene resource for crop breeding to control geminiviruses.

## 1. Introduction

Geminiviruses are obligate intracellular parasites that cause diseases in many economically important crops (e.g., tomato, corn, maize, cassava, and cotton); thus, they pose major threats to global food security. Geminiviruses have circular single-stranded DNA genomes that are encapsidated in twinned particles. Based on their genome organization, insect vector, and host range, geminiviruses can be classified into 14 genera, of which the genus *Begomovirus* is the largest [1]. Viruses in the genera *Becurtovirus*, *Capulavirus*, *Citlodavirus*, *Curtovirus*, *Eragrovirus*, *Grablovirus*, *Maldovirus*, *Mastrevirus*, *Mulcrilevirus*, *Opunvirus*, *Topilevirus*, *Topocuvirus*, and *Turncurtovirus* have monopartite genomes, whereas viruses in the *Begomovirus* genus have mono- or bipartite genomes. Monopartite begomoviruses contain six known open reading frames. The viral strand of the genome encodes the capsid protein V1 and V2, while the complementary strand encodes the C1/Rep, C2, C3, and C4 proteins [2]. Some monopartite begomoviruses are associated with satellite DNAs (α and β, each approximately 1.3–1.4 kb in length) and begomovirus/betasatellite complexes have caused numerous economically important diseases, including the earliest recorded plant viral disease [3,4,5]. Betasatellites are required for symptom expression in plants, although they depend on begomoviral DNA for replication and encapsidation [6,7].

Tomato yellow leaf curl virus (TYLCV) is a monopartite begomovirus. It is one of the most damaging and threatening viruses for tomato production worldwide. The selection of tomatoes that are resistant to the virus is an effective method for controlling disease caused by TYLCV. Currently, six TYLCV resistance genes (*Ty-1* to *Ty-6*) are known; some are used widely for introgression breeding [8]. *Ty-1*, *Ty-3*, *Ty-4*, and *Ty-6* are derived from the wild tomato species *Solanum chilense*, *Ty-2* is from *Solanum habrochaites*, and *ty-5* is from the commercial tomato cultivar Tyking [9,10,11,12,13,14,15,16,17,18,19]. *Ty-1*, *Ty-2*, *Ty-3*, *Ty-4*, and *Ty-6* are dominant resistance genes, while *ty-5* exhibits recessive inheritance. In recent years, *Ty-1*, *Ty-2*, *Ty-3*, and *ty-5* have been cloned. *Ty-1* and *Ty-3* are allelic and encode an RNA-dependent RNA polymerase [20]. *Ty-2* encodes a nucleotide-binding leucine-rich repeat protein that recognizes the Rep/C1 protein of TYLCV and induces a hypersensitive response to viral infection [21,22]. In susceptible tomatoes, *ty-5* encodes the messenger RNA surveillance factor Pelota [17,19]. The *ty-5* gene contains a single amino acid mutation, V16G, in Pelota (Pelota^V16G^); tomatoes that contain this mutation are resistant to TYLCV [17,19]. Until recently, it was unclear whether *ty-5* could confer effective resistance to other geminiviruses. *Ty-1* is a gene that encodes universal resistance to geminiviruses; however, it was recently reported that the resistance conferred by *Ty-1* is compromised by co-infection of TYLCV with a betasatellite [9,18]. No effective resistance gene is currently available for controlling begomovirus/betasatellite complexes. There is an urgent need to screen and identify new effective resistance genes to control the emerging begomovirus/betasatellite complexes. To our knowledge, no studies have investigated whether *ty-5* can confer effective resistance to begomovirus/betasatellite complexes.

In this study, we demonstrate that *ty-5* confers broad-spectrum resistance to various geminiviruses. *ty-5* confers effective resistance to two representative begomoviruses. The tomato *ty-5* line also exhibits partial resistance to beet curly top virus (BCTV), a virus in the genus *Curtovirus*. Importantly, *ty-5* confers resistance to infection by TYLCV with a betasatellite. Regardless of betasatellite status, significantly less DNA of these geminiviruses accumulates in the *ty-5* line than in the susceptible line. Moreover, knockdown of *Pelota* expression converts a *Nicotiana benthamiana* plant from a geminivirus-susceptible host to a geminivirus-resistant host. Our findings provide important insights concerning the use of *ty-5* to control geminiviruses.

## 2. Materials and Methods

### 2.1. Plant Materials and Virus Sources

Tomato (*Solanum lycopersicum*) and *N. benthamiana* plants were grown in an insect-free growth chamber at 25℃ under a 16-h light/8-h dark cycle. The *ty-5* tomato line, AVTO1227, was introduced from the World Vegetable Center in 2013 [17]; tomato Moneymaker was the susceptible line for TYLCV. Infectious clones of TYLCV Beijing isolate (MN432609) [23], BCTV (U02311.1) [24], and tomato leaf curl Yunnan virus Y194 isolate (TbLCYnV, AJ971265; Y194 refers to TbLCYnV) [25], as well as Y10 isolates of tomato yellow leaf curl China virus (TYLCCNV, AJ319675; Y10A refers to TYLCCNV DNA-A) and tomato yellow leaf curl China betasatellite (TYLCCNB, AJ421621; Y10β refers to TYLCCNB) [7], were previously described and have been maintained in our laboratory.

### 2.2. Agrobacterium-Mediated Inoculation and Disease Symptom Assessment

*S. lycopersicum* and *N. benthamiana* plants were agroinfected with the geminiviruses via *Agrobacterium tumefaciens*-mediated infiltration. *A. tumefaciens* EHA105 cultures were adjusted to an optical density of OD_600_ = 2.0 before infiltration into *S. lycopersicum* plants. For agroinfiltration of *N. benthamiana* plants, *A. tumefaciens* cultures were adjusted to an optical density of OD_600_ = 0.2. For viral infection analysis, the numbers of virus-infected plants with different disease symptom grades were counted and converted to percentages. Grades I to IV referred to plants that were asymptomatic, showed mild leaf curling symptoms, severe leaf curling symptoms, and severely curly leaves and stunting, respectively.

### 2.3. Total DNA Extraction, Southern Blotting, and Quantitative Polymerase Chain Reaction

Total DNA was isolated from infected young leaves of plants using the cetyltrimethylammonium bromide (CTAB) method, separated by 1% agarose gel electrophoresis, and then transferred to nylon membranes (Hybond N+; GE Healthcare, Pittsburgh, PA, USA). The membranes were hybridized at 55℃ with digoxigenin-labeled probes that had been prepared using a commercial kit (DIG High Prime DNA Labeling and Detection Starter Kit; Roche Diagnostics, Rotkreuz, Switzerland). The agarose gels were stained (Gel Stain; TransGen Biotech, Beijing, China) and used to confirm equal sample loading. Viral DNA accumulation was also detected by quantitative polymerase chain reaction (qPCR) using TB Green Premix Ex Taq II with 40 rounds of amplification (Takara, Japan). Viral DNA accumulation was normalized to the expression of 25S rRNA using the comparative Ct method (2^−ΔΔCt^), as previously described [26]. Primers used in this study are listed in Table S1. 

### 2.4. Plasmid Construction

For construction of a hairpin-based RNAi vector containing *NbPelota*, a partial fragment of *NbPelota* cDNA was amplified by PCR using the corresponding primers and cloned into the RNAi vector [27] by infusion; the reverse *NbPelota* fragment was cloned into the resulting vector using the restriction enzyme *Mlu*I and *Sal*I. The primers used in this study are listed in Table S1.

## 3. Results

### 3.1. Sequence Comparison of Pelota, the Candidate ty-5 Gene, among Tomato Lines

**Figure 1:**
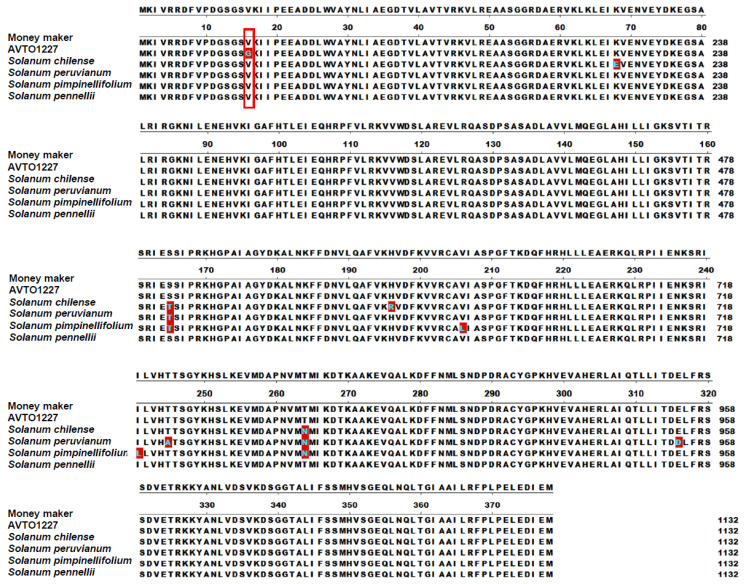
Alignment of the predicted amino acid sequences of *Pelota* genes from the susceptible tomato line Moneymaker, the resistant tomato line AVTO1227, *Solanum chilense*, *Solanum* *peruvianum*, *Solanum* *pimpinellifolium*, and *Solanum* *pennellii*. The difference between resistant and susceptible lines (valine vs. glycine at amino acid 16) is indicated with a red box. Residues that differ from Moneymaker are indicated in solid deep red.

The tomato line AVTO1227 exhibits effective resistance to TYLCV and the line Moneymaker is susceptible to TYLCV [17]. To identify nucleotide polymorphisms in the *Pelota* gene within the AVTO1227 resistant (R) line and susceptible (S) line, we cloned and sequenced the *Pelota* genes from both R and S lines. *Pelota* gene sequence comparisons revealed only one nucleotide difference, T47G, between the two lines. This mutation results in a single amino acid mutation, V16G, in the *Pelota* gene in the *ty-5* R line (Figure 1). We also examined this amino acid site in the Pelota protein in *S. chilense*, *S.* *peruvianum*, *S.* *pimpinellifolium*, and *S.* *pennellii*, and none of these wild-type tomatoes contained the V16G mutation (Figure 1). Thus, the sequencing results confirmed that the *ty-5* line of AVTO1227 specifically carries a V16G mutation in Pelota.

### 3.2. ty-5 Confers Resistance to Two Representative Begomoviruses in China

**Figure 2:**
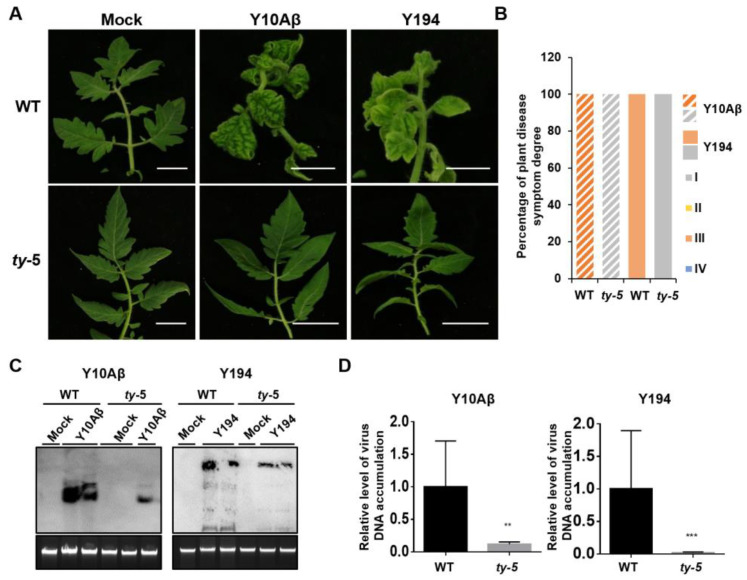
The *ty*-5 gene confers resistance to two representative begomoviruses from China. (**A**) Viral symptoms in the tomato *ty-5* line with a Pelota^V16G^ mutation and the wild-type (WT) line, agroinfected with either the pBinPLUS empty vector (mock), an infectious clone of tomato yellow leaf curl China virus (TYLCCNV)/tomato yellow leaf curl China betasatellite (TYLCCNB) (Y10Aβ), or tomato leaf curl Yunnan virus (TbLCYnV, Y194). The inoculated plants were photographed at 7 weeks post-inoculation (wpi). (**B**) The percentage and degree of plant disease symptoms in WT and *ty-5* plants infected by TYLCCNV/TYLCCNB (Y10Aβ) or TbLCYnV (Y194) at 7 wpi. In total, 15 plants from each line were used for the assays. Disease symptoms were classified from grade I (no symptoms) to grade IV (very severe symptoms). (**C**) Southern blotting analysis of viral DNA accumulation in WT and *ty-5* lines infected with TYLCCNV/TYLCCNB (Y10Aβ) or TbLCYnV (Y194) at 7 wpi. DNA fragments of TYLCCNV or TbLCYnV capsid protein were used as probes for detection of genomic DNA from TYLCCNV and TbLCYnV, respectively. (**D**) qPCR analysis of viral DNA accumulation in WT and *ty-5* lines infected with TYLCCNV/TYLCCNB (Y10Aβ) and TbLCYnV (Y194) at 7 wpi. Specific primers for TYLCCNV or TbLCYnV capsid protein were used to quantify the accumulation of viral DNA; 25S rRNA was used as the internal control. Asterisks denote statistically significant differences evaluated with Student’s *t* test, ** *p* < 0.01, *** *p* < 0.001.

To determine the resistance spectrum of *ty-*5, we used two representative begomoviruses from China (TYLCCNV/TYLCCNB and TbLCYnV). The tomato AVTO1227 line carrying the *ty-5* gene (hereafter referred to as the *ty-5* line) was inoculated with infectious clones of TYLCCNV/TYLCCNB or TbLCYnV by *Agrobacterium*-mediated infiltration. Plants agroinfiltrated with the pBinPLUS empty vector were used as mock controls. The tomato Moneymaker line (hereafter referred to as the WT line), in which TYLCV can induce strong disease symptoms, was also inoculated with the abovementioned infectious clones and used as controls. The disease symptoms of inoculated tomatoes were monitored at 3–7 weeks post-inoculation. Compared with mock controls, the Moneymaker WT line showed severe disease symptoms, including yellowing and leaf curling, at 3–7 weeks after inoculation with TYLCCNV/TYLCCNB (Y10Aβ) or TbLCYnV (Y194) (Figure 2A). However, the *ty-5* line inoculated with these two viruses remained symptomless. We then classified the disease symptoms from grade I (no symptoms) to grade IV (very severe symptoms; Figure S1). All inoculated plants in the WT line exhibited grade III symptoms, whereas no plant in the *ty-5* line exhibited any obvious disease symptoms (grade I; Figure 2B). Southern blotting analysis showed that the *ty-5* line accumulated significantly less genomic DNA from TYLCCNV/TYLCCNB (Y10Aβ) or TbLCYnV (Y194), compared with the WT line (Figure 2C). qPCR assays confirmed that *ty-5* inhibited the viral accumulation of these two begomoviruses in the resistant line (Figure 2D). These results suggest that *ty*-5 confers resistance to the two representative begomoviruses.

### 3.3. ty-5 Confers Resistance to Curtovirus

**Figure 3:**
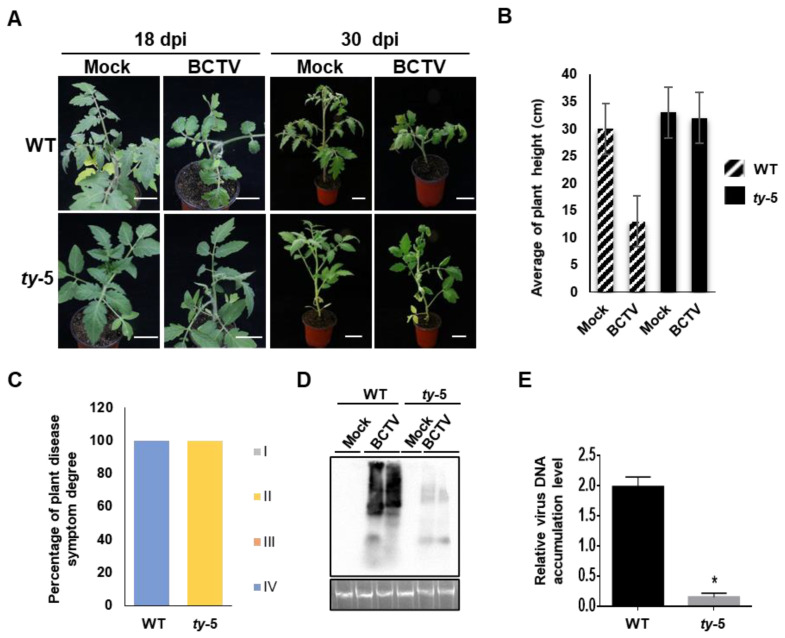
*ty*-5 confers resistance to beet curly top virus (BCTV), a geminivirus in the genus *Curtovirus.* (**A**) Viral symptoms of the tomato *ty-5* line and the wild-type (WT) line agroinfected with either the pBinPLUS empty vector (mock) or an infectious clone of BCTV. Systemically infected leaves and entire plants were photographed at 18 and 30 days post-inoculation (dpi), respectively. (**B**) The mean height of WT and *ty-5* plants infected with BCTV at 30 dpi. (**C**) The percentage and degree of disease symptoms in WT and *ty-5* plants infected with BCTV at 30 dpi. Fifteen plants from each of the WT and *ty-5* lines were used for the assays. Disease symptoms were classified from grade I (no symptoms) to grade IV (very severe symptoms). (**D**) Southern blotting analysis of viral DNA accumulation in WT and *ty-5* lines infected with BCTV at 30 dpi. A DNA fragment of BCTV capsid protein was used as a probe for detection of genomic DNA from BCTV. (**E**) qPCR analysis of viral DNA accumulation in WT and *ty-5* lines infected with BCTV at 30 dpi. Specific primers for BCTV capsid protein were used to quantify the accumulation of viral DNA; 25S rRNA was used as the internal control. Asterisks denote statistically significant differences evaluated with Student’s *t* test, * *p* < 0.05.

To further characterize the resistance spectrum of *ty-5*, we tested its resistance to the curtovirus BCTV. Both the *ty-5* and WT lines were inoculated with the infectious clone of BCTV or the pBinPLUS empty vector (mock control) by agroinfiltration. Compared to plants that were inoculated with the mock control, the WT line showed severe stunting at 18–30 days after BCTV infection (Figure 3A,B). However, the *ty-5* line inoculated with BCTV showed mild leaf curl symptoms and no stunting was observed at 18–30 days post-inoculation. We also classified the disease symptoms from BCTV into grades I (none) to IV (very severe). All inoculated plants in the WT line exhibited grade IV disease symptoms, while most plants in the *ty-5* line exhibited grade II disease symptoms (Figure 3C). Southern blotting analysis showed that significantly less BCTV genomic DNA accumulated in the *ty-5* line than in the WT line (Figure 3D). This finding was confirmed by qPCR (Figure 3E). Thus, the results suggest that *ty*-5 confers resistance not only to begomoviruses, but also to a curtovirus.

### 3.4. ty-5 Confers Resistance to TYLCV with Betasatellite

**Figure 4:**
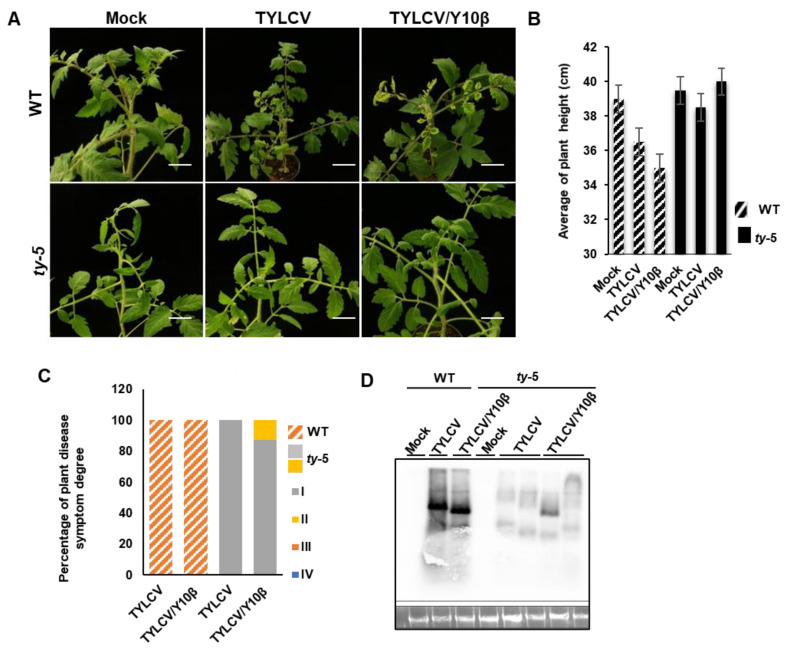
*ty-5* confers resistance to tomato yellow leaf curl virus (TYLCV) with a betasatellite. (**A**) Viral symptoms of the tomato *ty-5* line and wild-type (WT) line agroinfected with either the pBinPLUS empty vector (mock), an infectious clone of TYLCV, or infectious clones of TYLCV/tomato yellow leaf curl China betasatellite (Y10β). The mock- and virus-inoculated plants were photographed at 7 weeks post-inoculation (wpi). (**B**) The mean height of WT and *ty-5* plants infected with TYLCV, with and without a betasatellite, at 7 wpi. (**C**) The percentage of degree of disease symptoms in WT and *ty-5* plants infected by TYLCV, with and without a betasatellite at 7 wpi. Fifteen plants from each of the WT and *ty-5* lines were used for the assays. Disease symptoms were classified from grade I (no symptoms) to grade IV (very severe symptoms). (**D**) Southern blotting analysis of viral DNA accumulation in WT and *ty-5* lines infected by TYLCV or TYLCV/Y10β at 7 wpi. A DNA fragment of TYLCV capsid protein was used as a probe for detection of genomic DNA from TYLCV.

It has also been recently reported that the resistance of *Ty-1* to geminiviruses is compromised during co-infection by TYLCV with a betasatellite [9,18]. To test whether *ty-5* could confer resistance to TYLCV during co-infection with a betasatellite, the tomato *ty-5* line was agroinfiltrated with infectious clones of TYLCV alone, TYLCV/TYLCCNB, or a pBinPLUS empty vector (mock control). The WT line was also inoculated with TYLCV or TYLCV/TYLCCNB complex for comparison. Compared with plants that had been inoculated with TYLCV, the WT line showed severe disease symptoms after infection by the TYLCV/TYLCCNB (TYLCV/Y10β) complex (Figure 4A,B). TYLCV did not cause any obvious symptoms in the *ty-5* line. In the presence of a betasatellite, TYLCV induced no or mild leaf curl symptoms in the *ty-5* line (Figure 4A,B). Approximately 12.5% of the *ty-5* plants infected by the TYLCV/Y10β complex showed mild disease symptoms, whereas all others showed no symptoms (Figure 4C). In contrast, 100% of WT plants infected either by TYLCV or the TYLCV/Y10β complex showed severe disease symptoms (Figure 4C). Southern blotting analysis showed that substantial genomic DNA from TYLCV accumulated in the WT line, regardless of TYLCCNB status. Significantly less viral DNA accumulated in the *ty-5* line (Figure 4D). In the *ty-5* plant with mild leaf curl symptoms caused by the TYLCV/Y10β (Figure 4D), some viral DNA accumulated (the lane before the last lane); however, this was less than in the WT line infected with the TYLCV/Y10β. In the *ty-5* plant without leaf curl symptoms under infection by TYLCV/Y10β, the quantity of viral DNA (the last lane) was comparable to the quantity in the *ty-5* plant that was infected with TYLCV (Figure 4D).

### 3.5. Suppression of Pelota Expression in N. benthamiana Converts a Geminivirus-Susceptible Host to a Geminivirus-Resistant Host

**Figure 5:**
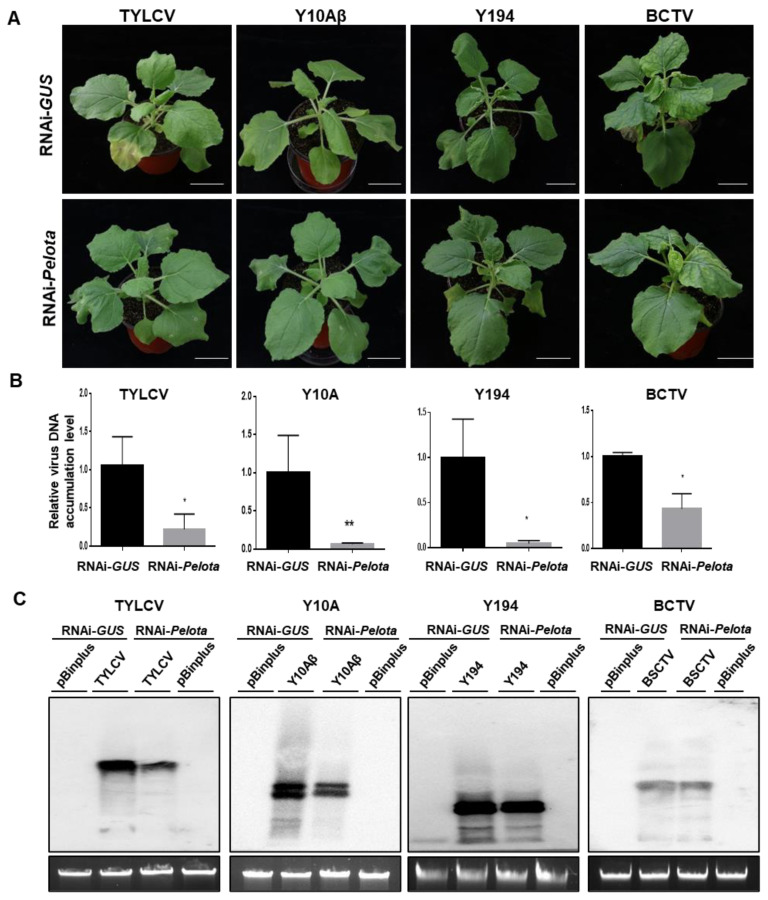
Knockdown of *Pelota* expression converts a *Nicotiana benthamiana* plant from a geminivirus-susceptible host to a geminivirus-resistant host. (**A**) Viral symptoms of *N. benthamiana* plants treated with agrobacterium that contains the construct expressing hairpin RNA targeting *Pelota* (RNAi-*Pelota*) or a hairpin RNA *GUS* control (RNAi-*GUS*), and then agroinoculated with either the pBinPLUS empty vector (mock), an infectious clone of tomato yellow leaf curl virus (TYLCV), tomato yellow leaf curl China virus/tomato yellow leaf curl China betasatellite (Y10Aβ), tomato leaf curl Yunnan virus (Y194), or beet curly top virus (BCTV). (**B**) qPCR analysis of viral DNA accumulation in geminivirus-infected RNAi-*Pelota*- and RNAi-*GUS*-treated plants. Leaf samples were collected from TYLCV, Y10Aβ, Y194, and BCTV-infected *N. benthamiana* plants at 9, 5, 9, and 12 dpi, respectively. Specific primers for TYLCV, Y10, Y194, or BCTV capsid protein were used to quantify the accumulation of viral DNA; 25S rRNA was used as the internal control. Asterisks denote statistically significant differences evaluated with Student’s *t* test, * *p* < 0.05, ** *p* < 0.01. (**C**) Viral DNA accumulation in geminivirus-infected RNAi-*Pelota*- and RNAi-*GUS*-treated plants was analyzed by Southern blotting. Leaf samples were collected from TYLCV, Y10Aβ, Y194, and BCTV-infected *N. benthamiana* plants at 12, 8, 12, and 9 dpi, respectively. DNA fragment of TYLCV, Y10, Y194, or BCTV capsid protein was used to quantify the accumulation of viral DNA.

*Pelota* with the V16G mutation confers resistance to geminiviruses. Thus, we tested whether knockdown of the *Pelota* expression of *N. benthamiana* plants would confer geminivirus resistance. *N. benthamiana* plants were treated with *Agrobacterium* that carries a construct expressing hairpin RNA targeting *Pelota* (RNAi-*Pelota*) or *Agrobacterium* that carries a control vector expressing hairpin RNA *GUS* gene (RNAi-*GUS*), and then agroinfected with TYLCV, TYLCCNV/TYLCCNB, TbLCYnV, or BCTV. Compared to the RNAi-*GUS*-treated control plant, the RNAi-*Pelota*-treated plant infected by TYLCV or TYLCCNV/TYLCCNB showed very mild or no disease symptoms (Figure 5A). Significantly less viral DNA accumulated systemically in the leaves of RNAi-*Pelota*-treated plants than in the leaves of RNAi-*GUS* control plants infected by TYLCV or TYLCCNV/TYLCCNB (Figure 5B,C). Similarly, RNAi-*GUS* control plants agroinfected with TbLCYnV exhibited severe disease symptoms in systemically infected leaves. In contrast, RNAi-*Pelota*-treated plants agroinfected with TbLCYnV showed mild disease symptoms (Figure 5A). As for BCTV, symptoms were similar between RNAi-*Pelota* and RNAi-*GUS* control plants (Figure 5A). Southern blotting analysis showed that less genomic DNA from TYLCV or TYLCCNV accumulated in RNAi-*Pelota* plants than in RNAi-*GUS* control plants, whereas no significant difference was found in terms of genomic DNA accumulation from TbLCYnV or BCTV (Figure 5C).

## 4. Discussion

In the present study, we demonstrated that *ty-5* confers broad-spectrum resistance to geminiviruses; it provided effective resistance to two representative begomoviruses present in China, TYLCCNV/TYLCCNB and TbLCYnV. *ty-5* also conferred partial resistance to BCTV, a virus in the genus *Curtovirus*. Finally, *ty-5* exhibited resistance to TYLCV co-infected with a betasatellite. Southern blotting and qPCR analyses showed that significantly less genomic DNA from these geminiviruses accumulated in the *ty-5* line than in the susceptible one. Moreover, knockdown of *Pelota* expression converted an *N. benthamiana* plant from a geminivirus-susceptible host to a geminivirus-resistant host.

In *Aedes aegypti*, Pelota deficiency suppresses Drosophila C virus capsid protein synthesis and dengue replication [28,29]. In tomatoes, Pelota with a V16G mutation confers resistance to the begomovirus TYLCV [17]. It was recently reported that a single-nucleotide polymorphism (A to G) located at the splice site of the ninth intron of *Pelota* in BaPep-5 pepper confers resistance to pepper yellow leaf curl Indonesia virus and pepper yellow leaf curl Aceh virus [30]. A single base substitution (T556A) in the coding sequence of *OsPelota* confers bacterial blight resistance by activating the salicylic acid pathway [31]. A *Pelota* mutant also confers resistance to rice blast [32]. Here, we found that *ty-5* with the V16G amino acid mutation in *Pelota* confers broad-spectrum resistance to various geminiviruses. Moreover, we found that knockdown of *Pelota* expression converted a *N. benthamiana* plant from a geminivirus-susceptible host to a geminivirus-resistant host. These results suggest that the *Pelota* gene is a good target for engineering to confer geminivirus resistance to various hosts. Geminiviruses cause diseases in many economically important crops, such as tomato, corn, maize, cassava, and cotton. Thus, *ty-5* is a promising resistance gene that can be targeted in various crops. *ty-5* only contains a single amino acid substitution, V16G, in Pelota. The introduction of this single amino acid change into Pelota might generate resistance lines in various crops. Although the necessary T to G gene editing cannot yet be conducted by the CRISPR/Cas9 method, future technology developments may enable sufficient editing for the conversion of geminivirus-susceptible crops to geminivirus-resistant crops.

Begomovirus/betasatellite complexes have recently emerged as causal agents for many economically important diseases. Many begomoviruses characterized in China are associated with betasatellites. We found that *ty-5* conferred resistance to TYLCCNV/TYLCCNB. Furthermore, TYLCV reportedly can associate with a betasatellite [8,9,33]. *Ty-1* is the most widely used resistance gene in tomato breeding. However, the resistance conferred by *Ty-1* is compromised by TYLCV upon co-infection with a betasatellite. This suggests that the TYLCV/betasatellite complex is able to overcome the widely used *Ty-1* resistance gene. However, our current findings indicate that the resistance conferred by *ty-5* cannot be compromised by co-replication of TYLCV with a betasatellite; thus, *ty-5* offers the potential to control this newly emerged TYLCV/betasatellite complex. 

Currently, we are not aware of how a single mutation in Pelota confers resistance to geminiviruses. In both animals and plants, Pelota proteins are reportedly involved in mRNA surveillance [34,35]. One possible scenario is that the mutant *Pelota^V16G^* may confer greater resistance to geminiviruses. The other scenario is that WT Pelota may be required for viral replication or transcription and the mutant Pelota^V16G^ may interfere with its ability to assist in viral replication or transcription. However, further efforts are required to dissect the role of Pelota in geminivirus infection. In conclusion, our findings suggest that *ty-5* can confer effective resistance to various geminiviruses. *ty-5* offers an important resistance gene resource for tomato crop breeding to control begomovirus/betasatellite complexes or other geminiviruses. Genome editing of *Pelota* also holds promise for the generation of geminivirus-resistant lines in other crops.

## Data Availability

All the data used in this study are already provided in the manuscript in the required section. There are no underlying data available.

## Supplementary Materials

The following supporting information can be downloaded at: www.mdpi.com/article/10.3390/v14081804/s1, Figure S1: Disease symptoms from grade I (no symptoms) to grade IV (very severe symptoms); Table S1: Primers used in this study.
