# Supplementary material for: ty-5 Confers Broad-Spectrum Resistance to Geminiviruses
Source: Viruses. 2022 Aug 17;14(8):1804. doi: 10.3390/v14081804 (PMC9415776; doi:10.3390/v14081804)
Supplement: Supplementary file 1 [file viruses-14-01804-s001.zip › viruses-1820728-supplementary.pdf]

viruses

MDPI

Supplemental Materials:

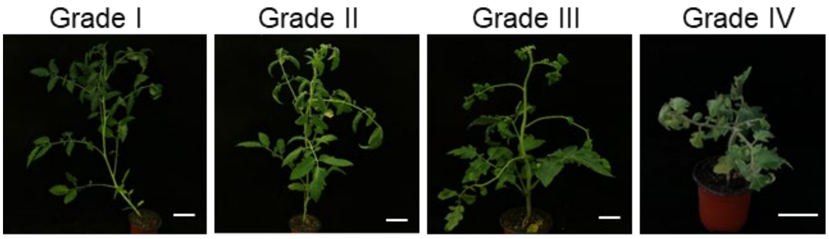

Figure S1. Disease symptoms from grade I (no symptoms) to grade IV (very severe symptoms).

Table S1. Primers used in this study.

**Purpose**	**Primers**	**Sequence (5'-3')**
To quantify the DNA titer of 25SRNA	Nb-25S RNA F	CCGAAGTTACGGATCCATTT
	Nb-25S RNA R	CCGAAGTTACGGATCCATTT
To quantify the DNA titer of TYLCV	qTYLCV-V1 F	CCGGAAGCCCAGAATATACAG
	qTYLCV-V1 R	ATCCCGATGCTCATAAGACTG
To quantify the DNA titer of Y10	qTYLCCNV-V1 F	AGAAGACAAATGTGGTCCAACAGG
	qTYLCCNV-V1 R	GCAATTAAAGACTTGTTGGAAATCCAT
To quantify the DNA titer of TbLCYnV	qTbLCYnV-V1 F	TGATGTTCCGAAGGGTTGTG
	qTbLCYnV-V1 R	AACCTCTTACCAACTCGATGC
To quantify the DNA titer of BCTV	qBCTV-V1 F	TGATATGTTGGGTGCTGGTG
	qBCTV-V1 R	AACTTCAAATGCTTCGTCACAG
To detect TYLCV	sTYLCV F	TGACTATGTCGAAGCGACCA
	sTYLCV R	CATTACAGCCTCAGACTGGT
To detect TbLCYnV	sTbLCYnV F	ATGTGGGATCCTTTACTCAACGAG
	sTbLCYnV R	TGTTGAAAATCCATCGGAGTACCAC
To detect TYLCCNV	sTYLCCNV F	ATGGCAGAAGGTGAGGAAATTCAGC
	sTYLCCNV R	TTAGAAGCATTTTCTGTGAACAATT
To detect BCTV	sBCTV F	ATGGATTTGGGCGGGAAACTTC
	sBCTV R	GCCAAACAAAGTGGCATACAT
To amplify the *Pelota* gene fragment, which was cloned into the RNAi vector using the infusion method	F-RNAi-Pelota F	ATCCGAGCTCGACGACAAGACCCGGGATCAGTTTCATCGTCACCTGAC
	F-RNAi-Pelota R	AGAAAATAATTATTTCCTTACCCCGGGCAGTAATGAGAAGTGTCTGG
To amplify the reversed *Pelota* gene fragment, which was cloned into RNAi vector using the *Mlu*I and *Sal*I sites	R-RNAi-Pelota F	ACGCGTCAGTAATGAGAAGTGTCTGG
	R-RNAi-Pelota R	GTCGACATCAGTTTCATCGTCACCTG
